# Refractory *Neoscytalidium dimidiatum* subcutaneous infection with dual CARD9/GATA2 variants: Synergistic clearance by voriconazole and targeted hyperthermia

**DOI:** 10.1016/j.jdin.2026.01.016

**Published:** 2026-02-12

**Authors:** Ying Huang, Xin Ran, Runyan Gao, Zhi Zhang, Jitong Sun, Sifen Lu, Yaling Dai, Yuping Ran

**Affiliations:** a Department of Dermatovenereology, West China Hospital, Sichuan University, Chengdu, China; b Laboratory of Dermatology, Clinical Institute of Inflammation and Immunology, Frontiers Science Center for Disease-related Molecular Network, West China Hospital, Sichuan University, Chengdu, China; c Precision Medicine Center, Breathing and Comorbidities Precision Medicine Key Laboratory of Sichuan Province, West China Hospital, Sichuan University, Chengdu, China; d Division of Clinical Microbiology, Department of Laboratory Medicine, West China Hospital, Sichuan University, Chengdu, China

**Keywords:** blackish-red dot sign, CARD9 mutation, dermoscopy, GATA2 mutation, *Neoscytalidium dimidiatum*, phaeohyphomycosis, scanning electron microscopy

*To the Editor:* We present a 51-year-old immunocompetent male carpenter with a 3-year history of multiple papules, nodules, and verrucous erythematous plaques on his neck, following accidental traumatic implantation of contaminated wood chips. The patient had hypertension but was otherwise healthy, with no tumors detected on screening. The lesion began as a small papule and slowly progressed. A previous diagnosis of fungal infection was made by biopsy at a local hospital, and the patient initially received oral itraconazole (200 mg/d) for 2 weeks with limited improvement.

Physical examination revealed multiple papules, nodules, and a well-demarcated erythematous plaque, measuring 8 × 4 cm on his neck. *In vivo* dermoscopy showed blackish-red dots on an erythematous base with yellowish scales (Supplementary Fig 1, available via Mendeley at https://data.mendeley.com/datasets/cgkjsg364m/1). Fungal culture and tissue metagenomic next-generation sequencing confirmed the diagnosis of cutaneous phaeohyphomycosis caused by *Neoscytalidium dimidiatum* (*N dimidiatum*). The patient was treated with itraconazole (400 mg/d) for 3 months, achieving satisfactory remission. Subsequently, he discontinued the treatment. He did not return to our hospital until the skin eruptions had relapsed and worsened (Fig 1) for 5 months. During the relapse, the patient resumed itraconazole therapy but showed no improvement.

**Fig 1:**
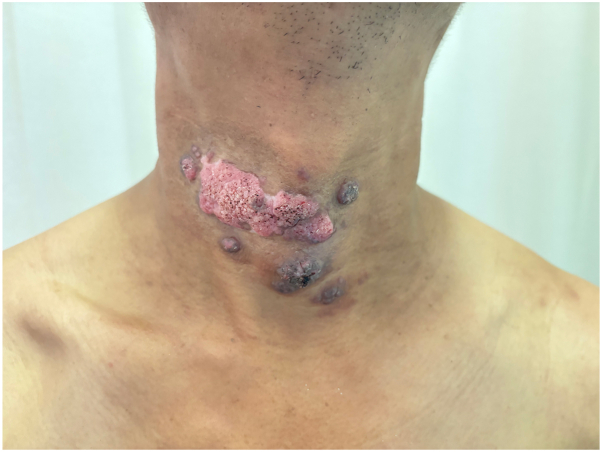
Phaeohyphomycosis presenting with multiple papules, nodules, and verrucous erythematous plaques on the neck when relapsed and worsened.

Given the chronic course and poor response, we performed whole-genome sequencing, which revealed a likely pathogenic heterozygous CARD9 variant c.1249C>T (p.Gln417∗) and a heterozygous GATA2 variant c.14C>T (p.Pro5Leu) of uncertain significance. We performed skin biopsy, tissue culture, and metagenomic next-generation sequencing again. Colonies initially appeared white, rapidly turning gray with a black reverse (Supplementary Fig 2, available via Mendeley at https://data.mendeley.com/datasets/cgkjsg364m/1). Slide culture (Supplementary Figs 3 and 4, available via Mendeley at https://data.mendeley.com/datasets/cgkjsg364m/1) and scanning electron microscopy (SEM) (Supplementary Fig 5, available via Mendeley at https://data.mendeley.com/datasets/cgkjsg364m/1) showed septate hyphae and ellipsoidal or oval-shaped, unicellular or bicellular arthroconidia in chains, which are the morphological characteristics of *Neoscytalidium spp*. Following dermoscopy-guided sampling of the blackish-red dots, we observed arthrospores via SEM at low and high continuous magnifications (Supplementary Fig 6, available via Mendeley at https://data.mendeley.com/datasets/cgkjsg364m/1). DNA extraction from cultured colonies identified the fungus as *N dimidiatum* (GenBank PQ272930). Dermoscopy-directed biopsy of the blackish-red dots showed diffuse mixed inflammatory cell infiltration in the dermis, accompanied by multinucleated giant cells (Supplementary Fig 7, available via Mendeley at https://data.mendeley.com/datasets/cgkjsg364m/1), and periodic acid-Schiff (Fig 2) and Gomori’s methenamine silver staining was positive (Supplementary Fig 8, available via Mendeley at https://data.mendeley.com/datasets/cgkjsg364m/1).

**Fig 2:**
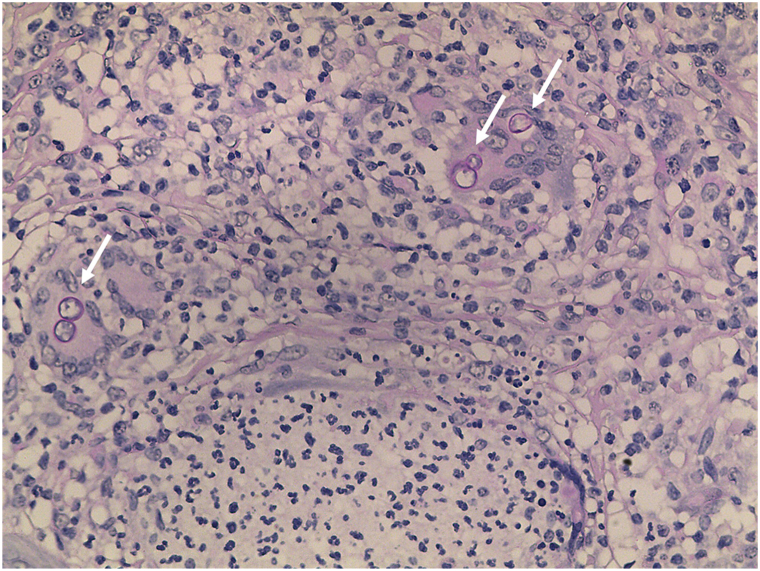
Fungal structures (*white arrow*) inside multinucleated giant cells by PAS staining (×400), biopsy consistent with phaeohyphomycosis. *PAS*, Periodic acid-Schiff.

Antifungal susceptibility testing indicated the isolate was sensitive to voriconazole (minimum inhibitory concentration: 0.06 μg/mL) and topical luliconazole. Additionally, temperature experiment showed the isolate did not grow at 42 °C. Based on these results, treatment was switched to oral voriconazole (400 mg/d), topical luliconazole cream, and hyperthermia using an electric heating pad (>42 °C, 60 minutes, once daily). After 7 months of treatment, the lesion was almost cured (Fig 3), with dermoscopic blackish-red dots fading (Supplementary Fig 9, available via Mendeley at https://data.mendeley.com/datasets/cgkjsg364m/1). No recurrence has been observed to date.

**Fig 3:**
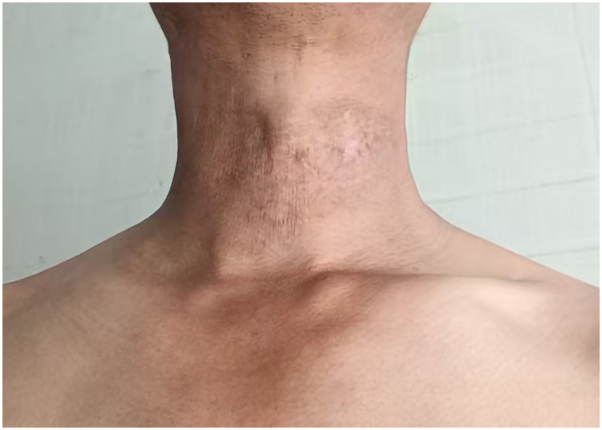
*N dimidiatum* subcutaneous infection. Significant improvement after antifungal treatment. *N dimidiatum*, *Neoscytalidium dimidiatum*.

Subcutaneous infections caused by *N dimidiatum* are infrequently documented.^1^ We present a case illustrating diagnostic and therapeutic challenges in managing such infections. Susceptibility gene screening identified 2 variants, in CARD9 and GATA2, both reported to be associated with increased fungal susceptibility, providing insight into susceptibility mechanisms and guiding individualized therapy for refractory fungal infections.^2^, ^3^, ^4^ In previous cases, blackish-red dots have been associated with subcutaneous fungal infections.^5^ In this case, the dermoscopic “blackish-red dot sign” observed in situ by SEM suggests that it may serve as a marker for early identification in chronic subcutaneous fungal infections.

## Conflicts of interest

None disclosed.
